# CCL2/CCR2 Expression in Locally Advanced Prostate Cancer and Patient Long-Term Outcome: 10-Year Results from the TROG 03.04 RADAR Trial

**DOI:** 10.3390/cancers16162794

**Published:** 2024-08-08

**Authors:** Mark Marsland, Chen Chen Jiang, Sam Faulkner, Allison Steigler, Kristen McEwan, Phillip Jobling, Christopher Oldmeadow, Brett Delahunt, James W. Denham, Hubert Hondermarck

**Affiliations:** 1 School of Biomedical Sciences and Pharmacy, College of Health, Medicine and Wellbeing, University of Newcastle, Callaghan, NSW 2308, Australia; mark.marsland@uon.edu.au (M.M.); chenchen.jiang@newcastle.edu.au (C.C.J.); sam.faulkner@newcastle.edu.au (S.F.); allison.steigler@newcastle.edu.au (A.S.); phillip.jobling@newcastle.edu.au (P.J.); 2 Hunter Medical Research Institute, University of Newcastle, New Lambton Heights, NSW 2305, Australia; 3 Department of Pathology and Molecular Medicine and Health Sciences, University of Otago, 6021 Wellington, New Zealand; 4 School of Medicine and Public Health, University of Newcastle, Callaghan, NSW 2308, Australia

**Keywords:** prostate cancer, CCL2, CCR2, cancer biomarker, RADAR trial

## Abstract

**Simple Summary:**

Prostate cancer is the second most common cancer among males and is a significant cause of morbidity and mortality. The chemokine C-C motif ligand 2 (CCL2) and its receptor C-C motif chemokine receptor 2 (CCR2) are expressed in prostate cancer but their prognostic value is unclear. This study investigated CCL2 and CCR2 as prognostic biomarkers in locally advanced prostate cancer using the large TROG 03.04 RADAR clinical trial cohort. The results demonstrate that despite being expressed in prostate tumours, CCR2 and CCL2 have no prognostic value in the disease.

**Abstract:**

This study investigated the prognostic value of the chemokine C-C motif ligand 2 (CCL2) and its receptor C-C motif chemokine receptor 2 (CCR2) expression in locally advanced prostate cancer treated with radiotherapy and androgen deprivation using the 10-year outcome data from the TROG 03.04 RADAR clinical trial. CCL2 and CCR2 protein expression in prostate cancer biopsies at the time of diagnosis were quantified by immunohistochemistry and digital quantification. CCR2 protein expression was detected in prostate cancer cells and was associated with prostate-specific antigen serum concentration (*p* = 0.045). However, neither CCL2 nor CCR2 tissue expression could predict prostate cancer progression, or other clinicopathological parameters including perineural invasion and patient outcome. In serum samples, CCL2 concentration at the time of diagnosis, as assayed by enzyme-linked immunosorbent assay, was significantly higher in patients with prostate cancer compared with benign prostatic hyperplasia (median difference 0.22 ng/mL, 95% CI, 0.17–0.30) (*p* < 0.0001) and normal controls (median difference 0.13 ng/mL, 95% CI, 0.13–0.17) (*p* < 0.0001). However, circulating CCL2 was not statistically significant as a predictor of disease progression and patient outcome. In conclusion, this study shows that although CCL2 and CCR2 are expressed in prostate cancer, with an increased level of CCL2 in the serum, neither CCL2 nor CCR2 expression has a clinical prognostic value in locally advanced prostate cancer.

## 1. Introduction

Prostate cancer is the second most common cancer among males and is a significant cause of morbidity and mortality [1]. The main difficulty in the management of prostate cancer is to determine the aggressiveness of the primary tumour at the time of diagnosis [2]. The vast majority of patients with prostate cancer are diagnosed with a clinically localized disease [3]. Although radical prostatectomy or radiation therapy is given with curative intent [4], 20–40% of patients will eventually experience biochemical recurrence (increase in serum prostate-specific antigen (PSA)) [5]; most patients will then receive androgen-deprivation therapy (ADT) which is the cornerstone in the management of advanced prostate cancer [6]. Biochemical recurrence following ADT, despite castrate levels of testosterone, represents the development of castration-resistant prostate cancer (CRPC), which, in many cases, is followed by metastatic disease [7]. Perineural invasion (PNI), a process in which cancer cells surround and invade nerves in the tumour microenvironment, has been associated with adverse prognosis [8] and the development of bone metastasis [9] in prostate cancer, but there is currently a lack of clinical prostate cancer prognostication. Thus, it is paramount to improve prostate cancer prognosis with new biomarkers to better stratify the disease and identify the most aggressive tumours at the time of diagnosis. 

In recent years, the chemokine CCL2 (C-C motif ligand 2), also known as monocytic chemotactic protein 1 (MCP-1), and its receptor CCR2 (C-C motif receptor 2) have gained attention for their association with cancer occurrence and therapeutic resistance [10,11,12]. Beyond their roles as mediators of immune cell recruitment and inflammation [13], accumulating evidence suggests that dysregulated CCL2/CCR2 signalling is intricately linked to androgen receptor (AR) signalling and the development of CRPC [14]. Preclinical studies have demonstrated that androgen deprivation induces the upregulation of CCL2 expression in prostate cancer cells, fostering tumour growth and metastasis through the recruitment of tumour-associated macrophages (TAMs) [15]. Moreover, clinical investigations have suggested associations between elevated CCL2 serum concentration and adverse clinicopathological features, including higher Gleason scores, advanced disease stage, and increased risk of biochemical recurrence following ADT [10,16]. Similarly, CCR2 expression has been implicated in promoting tumour cell survival, proliferation, and metastasis, contributing to treatment resistance and poor prognosis in CRPC [17]. However, the value of CCL2 and CCR2 as prognostic biomarkers in prostate cancer is unclear and warrant further investigation [18].

The TROG 03.04 Randomised Androgen Deprivation and Radiotherapy (RADAR) Trial (NCT00193856) is a phase III multicentre clinical trial for locally advanced prostate cancer. Using a 2 × 2 factorial design, it investigated the effect of adjuvant androgen deprivation and 18 months of zoledronic acid in association with definitive radiation treatment on biochemical (PSA) control and patient survival. A total of 1071 men with histological confirmation of primary prostate cancer grade, baseline eligible T stages, and PSA were recruited during the period 2003–2007. Tumour biopsies and blood samples were collected at the time of diagnosis. Data on PSA and clinical progressions, as well as survival, were recorded up to trial closeout in 2017. The findings of the trial have been reported, in particular the 6.5- [19] and 10-year [20] endpoint outcomes. 

In the present study, we used the TROG 03.04 RADAR clinical trial to clarify the prognostic value of CCL2/CCR2 expression in locally advanced prostate cancer. CCL2 and CCR2 protein expression was studied by immunohistochemistry in biopsies, and by enzyme-linked immunosorbent assay (ELISA) in blood samples, obtained from patients at the time of diagnosis. Our results show that CCL2 and CCR2 are expressed in prostate cancer cells, with CCL2 increased in the serum of prostate cancer patients, but the expression of both CCL2 and CCR2 exhibited no prognostic value. 

## 2. Materials and Methods

### 2.1. Prostate Cancer Patient Cohort

This study was conducted according to the Declaration of Helsinki and approved by the Human Research Ethics Committee at The University of Newcastle, Australia (X11-0023 and H-2012-0063). The cases were from the histological component of the Trans-Tasman Radiation Oncology Group TROG 03.04 RADAR (Randomised Androgen Deprivation And Radiotherapy) trial. This was a phase 3 trial that recruited 1071 subjects between October 2003 and August 2007 from 23 treatment centres in New Zealand and Australia. The trial’s enrolment eligibility criteria were men ≥ 18 years of age with histologically confirmed adenocarcinoma of the prostate without lymph node or systemic metastases. Tumours were of any ISUP (International Society of Urological Pathology) grade and baseline serum PSA level, of clinical staging category cT2b to cT4, or clinical staging category cT2a with ISUP grade > 1 and baseline PSA ≥ 10 ng/mL. The trial’s design and outcomes have been previously described [19,20]. In short, diagnostic thin core biopsies were retrieved for all patients and reviewed blind by the Trial Pathologist. The grading of tumours was performed according to the recommendations of the ISUP consensus conference, and an ISUP grade was assigned to each case. Assessment of PNI was performed on all cores. Additionally, in a sub-study of the trial, serum was collected at time of diagnosis from RADAR patients at the highest recruiting site; these samples were graded by the pathologist at the recruiting site and are referred to as institutional. Serum samples from patients with benign prostatic hyperplasia (BPH) were provided by The Australian Prostate Cancer Bio-resource (APCB) and were age-matched. Control samples were provided by The Hunter Community Study (HCS), a longitudinal cohort study [21]. Our study used 386 sera from age-matched men, excluding those with any form of cancer, history of obstructive urinary symptoms, prostatectomies, or prostatitis.

### 2.2. Immunohistochemistry 

Tissue biopsy slides were processed for 3′,3′-diaminobenzidine (DAB) immunohistochemistry using a Ventana Discovery Ultra (Roche, Indianapolis, IN, USA) by the TRI Histology Core Facility for CCL2 and by the Hunter Cancer Biobank for CCR2. Sections were labelled for anti-CCL2 (1:200, Thermo Fisher Scientific, North Ryde, NSW, Australia, #MA5-17040) and anti-CCR2 (1:500, clone 48607, R&D Systems #MAB150). All further steps of the immunohistochemistry process have previously been described by our laboratory [22]. 

### 2.3. Digital Quantification of Immunohistochemistry

Following IHC staining, sample slides were digitised as previously described by our laboratory [23]. Quantification of immunohistochemistry analyses were performed using the HALO^TM^ image analysis platform (version 3.3, Indica Labs, Albuquerque, NM, USA). To differentiate tissues and pixel intensity values corresponding to DAB staining tissue, classification algorithms were used and calculated using the Cytonuclear module [24], which detected and quantified protein expression in the cytoplasm. Pixel intensity values were then used to determine the H-scores for each core. The H-score reflects the percentage of total cells with strong, moderate, and weak positive staining in the cytoplasm and is calculated by using the following formula: H-Score = (1 × % weak stain positive cells) + (2 × % moderate stain positive cells) + (3 × % strong stain positive cells). 

### 2.4. Enzyme-Linked Immunosorbent Assay (ELISA)

Detection and quantification of CCL2 in the serum of prostate cancer patients was performed using a sandwich ELISA kit (Cat No. BEK-2059) from Biosensis Pty Ltd., (Thebarton, SA, Australia). This assay uses a sensitive and specific monoclonal human CCL2 antibody precoated onto a 96-well strip plate, a biotinylated detection antibody, and an enzyme Avidin-Biotin-Peroxidase complex (ABC). Finally, the peroxidase substrate TMB is added to induce a coloured reaction product. All serum samples were tested at 1:10 dilution, and assay kits were used as per the manufacturer’s instructions. Validation of the results was performed as previously described by our laboratory [25]. Plates were read with a Spectramax plate reader (Molecular Devices LLC, San Jose, CA, USA, M3). Optimisation of the TMB incubation step was performed with every ELISA experiment, as previously described [25]. 

### 2.5. Statistical Analyses

Patient characteristics were summarised with frequencies and percentages for categorical variables or with median and interquartile range (IQR: 25th–75th percentiles) for continuous variables. Each biomarker was analysed as a continuous, non-normally distributed variable. Associations between the biomarker and clinicopathological variables were assessed using Wilcoxon rank-sum or Kruskal–Wallis tests for categorical variables and Spearman’s rank correlation for continuous variables. Uni- and multivariable Cox regression (statistical method applied when there are multiple, potentially interacting covariates) analyses were performed to measure the prognostic impact of the biomarker on time-to-event outcomes. Multivariable models were adjusted for baseline prognostic and treatment factors including age, ISUP grade, clinical T-stage, PSA, AS duration, use of zoledronic acid, and RT dose. In these models, the Fine and Gray statistical method was used to account for competing risks. The competing risk for PSA progression (PSA rise of 2 ug/L above post-treatment level), distant progression, and bone progression was death due to any cause, and for prostate cancer-specific mortality, was other cause of death. In competing risks models, the proportional hazard assumption was tested by including interaction terms between each variable and time; if the associated variable violated the assumption, the interaction term was retained in the model. In the Cox regression model, Schoenfeld residuals was used to assess proportional hazards for all-cause mortality. In this model, covariates that violated the assumption were stratified for. All *p* values were two-sided, and statistical significance was defined as *p* < 0.05. Statistical analyses were calculated using Stata/IC Version 15.1 (StataCorp LLC, College Station, TX, USA).

## 3. Results

### 3.1. Detection of CCR2 Protein in Prostate Cancer Biopsies

**Figure 1:**
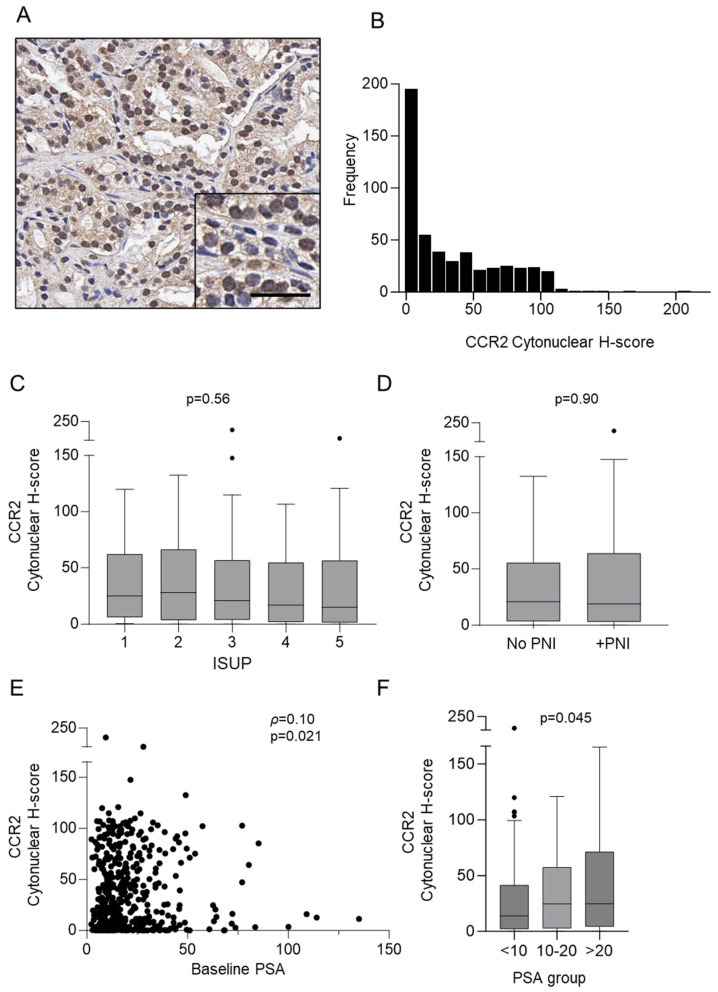
CCR2 expression in prostate cancer core biopsies. (**A**) Representative picture for the immunohistochemical detection of CCR2. Scale bar = 25 µm. (**B**) Digital quantification of CCR2 expression showing staining H-score distribution (H-score = 0–207.3); most samples with positive CCR2 staining at low intensity (median H-score = 20.17, IQR 3.08–58.17). (**C**) CCR2 staining intensities according to grouped pathological subtypes: ISUP 1 (median H-score = 28.18, IQR 3.59–63.39), ISUP 2 (median H-score = 20.99, IQR 2.63–61.16), ISUP 3 (median H-score = 14.92, IQR 1.80–56.99), ISUP 4 (median H-score = 20.38, IQR 6.21–46.10), and ISUP 5 (median H-score = 15.09, IQR 2.90–64.11). (**D**) CCR2 expression according to PNI-negative (no PNI) (median H-score = 21.66, IQR 3.23–56.29) and PNI-positive (+PNI) (median H-score = 18.71, IQR 2.84–63.81) status. (**E**) Correlation of CCR2 expression and baseline PSA levels (ρ = 0.10, *p* = 0.021). (**F**) CCR2 staining intensities according to grouped PSA levels: <10 (median H-score = 13.94, IQR 2.210–41.47), 10–20 (median H-score = 24.61, IQR 2.775–57.43), and >20 (median H-score = 24.71, IQR 4.355–71.24). Data are expressed as medians (horizontal line in the centre of the box), and box limits indicate the interquartile range (IQR) with the whiskers extending 1.5 times the IQR from the 25th and 75th percentiles; outliers are represented by dots. H-score distributions were compared using the Wilcoxon rank-sum (dichotomous) or Kruskal–Wallis. ISUP, International Society of Urological Pathology; PNI, perineural invasion.

Staining of CCR2 by immunohistochemistry was performed on all core biopsy tissue positive for prostate cancer (n = 501); baseline characteristics of patient samples are shown in Table 1 and the results are presented in Figure 1 and Table 2. CCR2 was observed in 481/501 (96%) of samples, and representation of CCR2 staining is shown in Figure 1A. Observation of CCR2 staining revealed a wide range of stain intensity, with most at a lower intensity. We confirmed our observations by digital quantification and found that while there was a wide range of CCR2 staining (H-score = 0–207.3), most CCR2-positive samples were at low intensity (median H-score = 20.17, IQR 3.08–58.17) (Figure 1B). There was also no significant difference in CCR2 expression between ISUP grades (Figure 1C). Investigation of PNI-positive vs. PNI-negative samples found no significant difference between H-scores of PNI-positive and PNI-negative samples (Figure 1D). We wanted to know if there was an association between CCR2 tissue expression and baseline PSA levels in the blood. Our results revealed there was a significant, but very weak, positive correlation between CCR2 and PSA (ρ = 0.10, *p* = 0.021) (Figure 1E). Furthermore, when we grouped the PSA levels (<10, 10–20, and >20) we found that CCR2 was increased as the PSA groups increased (*p* = 0.045) (Figure 1F). We then investigated the association of CCR2 protein in prostate cancer patient tissue with time-to-event outcomes by performing univariable and multivariable analyses with 10-year endpoint data from the TROG 03.04 RADAR trial (Table 2). CCR2 expression was not statistically significantly associated with disease progression (PSA or clinical) or mortality outcome.

### 3.2. Detection of CCL2 Protein in Prostate Cancer Core Biopsies

**Figure 2:**
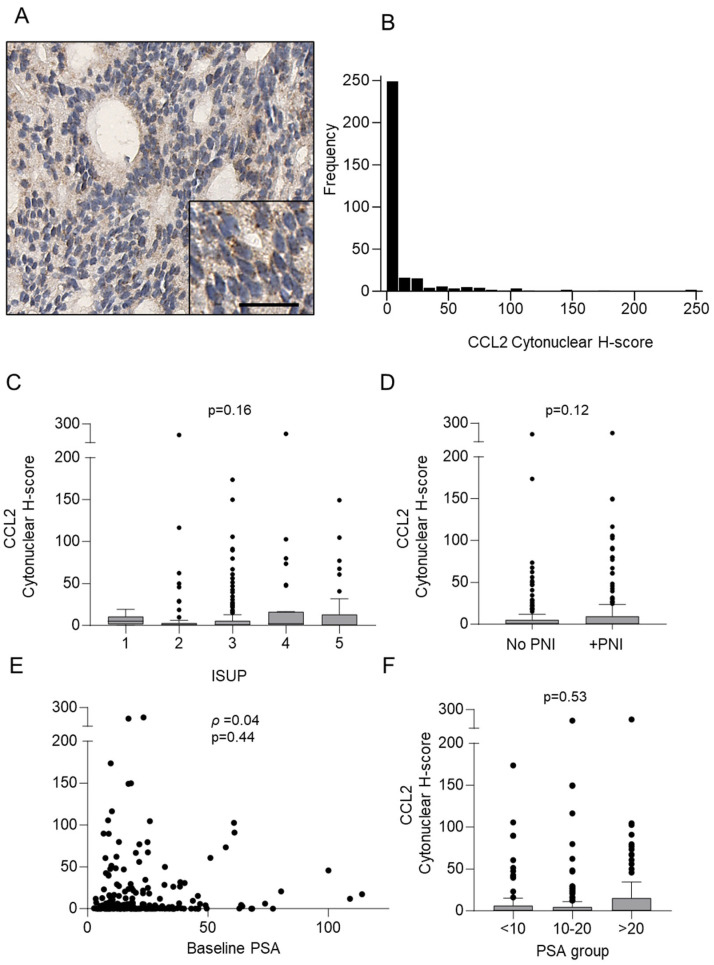
CCL2 expression in prostate cancer core biopsies. (**A**) Representative picture for the immunohistochemical detection of CCL2. Scale bar = 25 µm. (**B**) Digital quantification of CCL2 expression showing staining H-score distribution (H-score = 0–247.5) with most staining at low intensity (median H-score = 0.648, IQR 0.062–5.706). (**C**) CCL2 expression according to grouped pathological subtypes: ISUP 1 (median H-score = 1.06, IQR 0.15–4.57), ISUP 2 (median H-score = 0.733, IQR 0.10–5.18), ISUP 3 (median H-score = 0.35, IQR 0.00–2.40), ISUP 4 (median H-score = 0.66, IQR 0.20–15.96), and ISUP 5 (median H-score = 0.25, IQR 0.00–16.45). (**D**) CCL2 expression according to PNI-negative (no PNI) (median H-score = 0.74, IQR 0.19–5.33) and PNI-positive (+PNI) (median H-score = 0.43, IQR 0.00–9.47) status. (**E**) Correlation of CCL2 expression and baseline PSA levels (ρ = 0.04, *p* = 0.44). (**F**) CCL2 staining intensities according to grouped PSA levels: <10 (median H-score = 0.7762, IQR 0.00–6.323), 10–20 (median H-score = 0.5756, IQR 0.055–4.782), and >20 (median H-score = 0.6472, IQR 0.0752–15.23). Data are expressed as medians (horizontal line in the centre of the box), and box limits indicate the interquartile range (IQR) with the whiskers extending 1.5 times the IQR from the 25th and 75th percentiles; outliers are represented by dots. H-score distributions were compared using the Wilcoxon rank-sum (dichotomous) or Kruskal–Wallis. ISUP, International Society of Urological Pathology; PNI, perineural invasion.

Staining of CCL2 by immunohistochemistry was performed on core biopsy tissue positive for prostate cancer (n = 314); baseline characteristics of patient samples are shown in Table 3 and the results are presented in Figure 2 and Table 4. CCL2 was observed in 237/314 (75.5%) of samples, and representation of CCL2 staining is shown in Figure 2A. Observation of CCL2 staining revealed a wide range of stain intensity; however, most samples appeared to have low-intensity CCL2 staining. We confirmed our observations by digital quantification and found that while there was a wide range of CCL2 staining (H-score = 0–247.5), most staining was at low intensity (median H-score = 0.648, IQR 0.062–5.706) (Figure 2B). We wanted to see if there was a difference in CCL2 expression between ISUP grades and found that there was no significant difference in the median H-scores between ISUP grades (Figure 2C). Interestingly, however, ISUP 1 had the lowest range of CCL2 stain intensity (H-score = 0–19.16), compared to ISUP 2 (H-score = 0–240.9), ISUP 3 (H-score = 0–173.7), ISUP 4 (H-score = 0–247.5), and ISUP 5 (H-score = 0–149.3). We then investigated if there was a difference in PNI-positive vs. PNI-negative samples. There was no significant difference between the median H-score of PNI-positive and PNI-negative samples (Figure 2D). We wanted to know if there was an association between CCL2 tissue expression and baseline PSA levels in the blood. Our results revealed that there was no significant correlation between CCL2 and PSA (Figure 2E), and when we grouped the PSA levels (<10, 10–20, and >20), we found no significant difference in CCL2 levels between groups (Figure 2F). We then investigated the association of CCL2 protein expression with time-to-event outcomes by performing univariable and multivariable analysis with 10-year endpoint data (Table 4). The data showed that CCL2 expression was not associated with disease progression (PSA or clinical) or mortality outcome.

### 3.3. CCL2 Serum Concentration in Patients with Prostate Cancer Compared to Normal and Benign Prostatic Hyperplasia

**Figure 3:**
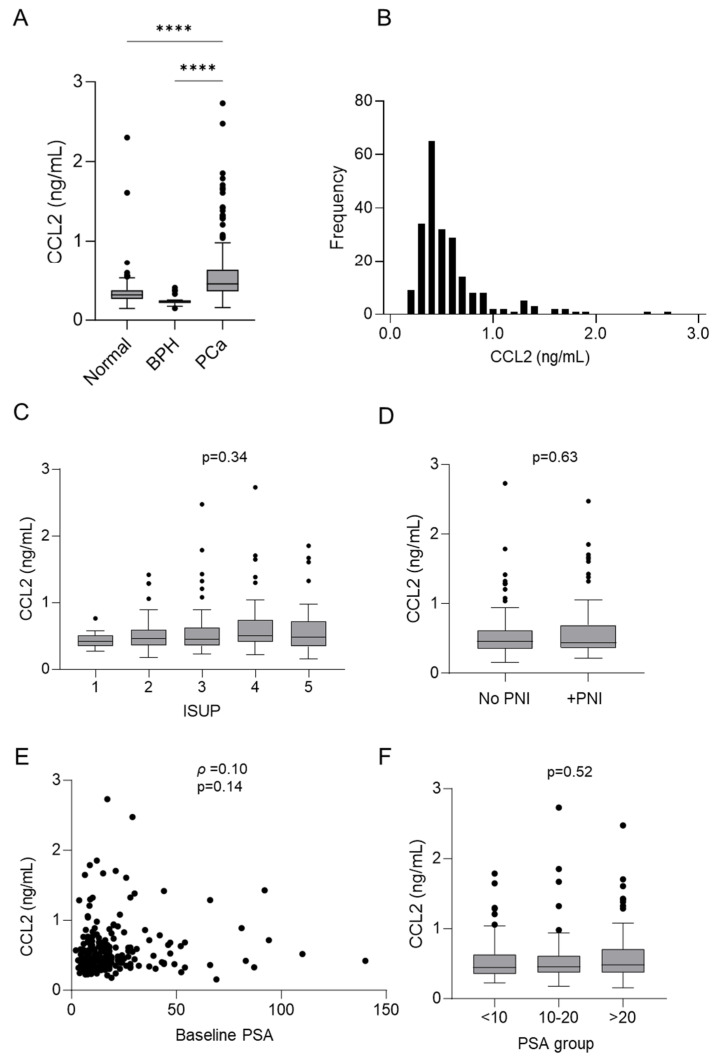
CCL2 serum concentration in patients with prostate cancer compared to normal and benign prostatic hyperplasia. Circulating CCL2 in the serum of patients at the time of tumour diagnosis was measured by ELISA. (**A**) The median CCL2 serum concentration was 0.45 ng/mL (IQR 0.36–0.63) compared to BPH serum (0.23 ng/mL, IQR 0.22–0.25) (median difference 0.22 ng/mL, 95% CI 0.17–0.30) (*p* < 0.0001) and normal serum (0.32 ng/mL, IQR 0.27–0.37) (median difference 0.13 ng/mL, 95% CI 0.13–0.17) (*p* < 0.0001). (**B**) CCL2 was detected in all samples as shown by the distribution graph. (**C**) CCL2 median concentration according to grouped pathological subtypes: ISUP 1 0.42 ng/mL (IQR 0.36–0.49), ISUP 2 0.46 ng/mL (IQR 0.36–0.60), ISUP 3 0.45 ng/mL (IQR 0.35–0.61), ISUP 4 0.51 ng/mL (IQR 0.41–0.74), and ISUP 5 0.48 ng/mL (IQR 0.34–0.72). (**D**) CCL2 median concentration according to PNI-negative (no PNI) 0.46 ng/mL, IQR 0.36–0.60) and PNI-positive (+PNI) 0.44 ng/mL (IQR 0.37–0.69) status. (**E**) Correlation of CCL2 concentration and baseline PSA levels (ρ = 0.10, *p* = 0.14). (**F**) CCL2 median concentration according to grouped PSA levels: <10 0.45 ng/mL (IQR 0.35–0.63), 10–20 0.46 ng/mL (IQR 0.38–0.61), and >20 0.48 ng/mL (IQR 0.37–0.70). **** *p* < 0.0001; BPH, benign prostatic hyperplasia; ISUP, International Society of Urological Pathology; PNI, perineural invasion.

The circulating level of CCL2 in the serum of prostate cancer patients at the time of diagnosis (n = 220) was measured by ELISA. The baseline characteristics of patient samples are shown in Table 5. First, we compared these measurements to CCL2 concentration in patients with benign prostatic hyperplasia (BPH) (n = 20) and with normal noncancer serum (n = 386). The results are shown in Figure 3 and Table 6. CCL2 was detected in all samples (Figure 3A,B). The median CCL2 concentration in prostate cancer serum was 0.45 ng/mL (IQR 0.36–0.63) and was significantly higher compared to BPH serum (0.23 ng/mL, IQR 0.22–0.25) (median difference 0.22 ng/mL, 95% CI, 0.17–0.30) (*p* < 0.0001) and normal serum (0.32 ng/mL, IQR 0.27–0.37) (median difference 0.13 ng/mL, 95% CI, 0.13–0.17) (*p* < 0.0001). We then investigated if there was a difference in CCL2 concentration between prostate cancer ISUP grades (Figure 3C). The median CCL2 concentrations of ISUP grades were ISUP 1 (n = 10) 0.42 ng/mL (IQR 0.36–0.49), ISUP 2 (n = 60) 0.46 ng/mL (IQR 0.36–0.60), ISUP 3 (n = 59) 0.45 ng/mL (IQR 0.35–0.61), ISUP 4 (n = 48) 0.51 ng/mL (IQR 0.41–0.74), and ISUP 5 (n = 43) 0.48 ng/mL (IQR 0.34–0.72). PNI was explored next (Figure 3D), and our results showed that patients with positive PNI status (n = 78) had a median CCL2 concentration of 0.44 ng/mL (IQR 0.37–0.69) compared to patients without PNI (n = 140) (0.46 ng/mL, IQR 0.36–0.60); therefore, there was no significant difference in CCL2 concentration between cancer of different grades or between PNI-positive or -negative status. We wanted to know if there was an association between circulating CCL2 and baseline PSA levels in the blood. Our results revealed there was no significant correlation between CCL2 and PSA (Figure 3E), and when we grouped the PSA levels (<10, 10–20, and >20) we found no significant difference in CCL2 levels between groups (Figure 3F). We then investigated the association of CCL2 concentration in prostate cancer patient serum with time-to-event outcomes by performing univariable and multivariable analysis with 10-year endpoint data from the TROG 03.04 RADAR trial (Table 6). Univariate analysis showed that, at 10 years, PSA progression occurred in 90 men (41%) and was associated with elevated CCL2 concentration (sub-hazard ratio (sHR) = 1.50; *p* = 0.017). However, when using multivariable analysis, the hazard ratio was reduced (sHR = 1.19; *p* = 0.35). CCL2 serum concentration in prostate cancer patients was not statistically significantly associated with clinical progression or mortality outcomes (Table 6).

### 3.4. Correlation of CCL2/CCR2 Expression in Prostate Cancer Patients

The overlap between the cohorts used for the quantification of CCL2 in serum, CCL2 in tissue, and CCR2 in tissue is presented in Supplementary Figure S1 (Venn diagram in Figure S1A). There appeared to be a slightly positive correlation between CCL2 tissue H-score and CCL2 serum concentration (Figure S1B), but this did not reach statistical significance (n = 26, *p* = 0.40). Interestingly there was a statistically significant positive correlation between CCR2 tissue H-score and CCL2 serum concentration (Figure S1C) (n = 29, *p* = 0.0149). Between CCL2 and CCR2 expression, we found a slightly positive relationship (n = 289, *p* = 0.0156) (Figure S1D).

## 4. Discussion

Our study examined the expression of CCR2 and CCL2 protein in locally advanced prostate cancer and found that they have no prognostic value in the disease. In contrast, other studies have suggested a prognostic value for CCR2 [26] and CCL2 [10,27,28] with prostate cancer progression. Below, we discuss how biological and technical differences could explain these discrepancies. 

CCR2 protein expression in prostate cancer biopsies was detected in most cases, and we found no correlation with clinicopathological parameters and prostate cancer progression or patient outcome. A previous study suggested that CCR2 expression was correlated with Gleason score and clinicopathological staging [26]. However, the number of prostate cancer patients included in this previous study was limited (n = 96), not targeted to locally advanced tumours, and microarrays were used for immunohistochemical detection of CCR2. Also, they did not follow patient outcomes and survival. In contrast, our study included a larger patient cohort (n = 501), was focused on locally advanced tumours, used biopsies for immunohistochemistry, and investigated 10-year patient follow-up. Therefore, our study had greater statistical power and clinical relevance, and highlighted that CCR2 expression has no significant prognostic value in locally advanced prostate cancer.

CCL2 protein expression in prostate cancer biopsies was detected in the majority of cases, and no correlation was found with clinicopathological parameters and prostate cancer progression or patient outcome. CCL2 was previously shown to act as a paracrine and autocrine growth factor for prostate cancer cells, and in a small cohort of 83 prostate cancers, it was suggested that CCL2 tissue expression was associated with advanced pathologic stage; however, the study did not include any data on tumour progression or patient outcome [27]. Another study including a patient cohort of 41 prostate cancers previously reported CCL2 tissue expression in ~50% of cases, with patient outcome significantly worse with lower survival time in patients with CCL2 overexpressing tumours [28]. However, in the locally advanced prostate cancer setting, with a cohort of 314 prostate tumours, we found no association between CCL2 protein expression and tumour progression or patient outcome. 

In terms of circulating levels of CCL2, it has previously been suggested that circulating CCL2 level in the blood is increased in prostate cancer and is a predictor of prostate cancer progression [10,16]. In this present study, we confirmed that circulating CCL2 concentration was significantly higher in patients with prostate cancer compared with BPH and normal controls. However, our study also showed that despite an increased level of CCL2 in prostate cancer, circulating CCL2 could not predict disease progression or patient outcome.

To date, and despite its limitations, PSA is the most significant biomarker in prostate cancer [1]. We found that CCR2 tissue expression in prostate tumours was associated with PSA blood level. On the other hand, CCL2 tissue expression was not associated with PSA blood level. Similarly, no correlation was found between circulating CCL2 and PSA levels. Interestingly a previous study found that in 30 out of 41 prostate cancer patients, PSA values in CCL2 positive patients were significantly higher than in CCL2 negative patients, but when taking into account the entire cohort, they found no significant association [28]. Here, we confirmed the absence of association between circulating levels of CCL2 and PSA in prostate cancer. 

During PNI, prostate cancer cells are able to grow alongside and invade prostatic nerves [8]. An association between PNI and prostate cancer progression was previously demonstrated, with PNI increased in high-grade tumours [9,29]. Importantly, in the TROG 03.04 RADAR trial cohort, PNI at the time of diagnosis was found to be associated with the future development of bone metastasis [9]. Interestingly, there are reports of CCR2 and CCL2 playing a potential role in stimulating PNI [30,31]. In cervical cancer, it was suggested that CCR2 expression was increased in patient samples with PNI compared to the non-PNI groups [31]. In prostate cancer, it was shown that the expression of CCR2 in a prostate cancer cell line correlated with cell migration towards dorsal root ganglion that expressed CCL2 [30]. Thus, despite experimental data suggesting a role for CCR2 and CCL2 in PNI [30], in this cohort of locally advanced prostate cancer, we found no significant change in CCR2 or CCL2 protein expression in prostate cancer patients in relation to PNI. 

## 5. Conclusions

In conclusion, using the large TROG 03.04 RADAR clinical trial cohort, our study has clarified the clinical significance of CCR2 and CCL2 protein expression in locally advanced prostate cancer by showing that despite being expressed in prostate tumours, CCR2 and CCL2 have no prognostic value in the disease. 

## Figures and Tables

**Table 1:** Clinicopathological characteristics of patients used for CCR2 detection (n = 501).

Characteristic		
Age, median (IQR), years	68.7	(63.9–73.1)
PSA, median (IQR), ng/mL	15.2	(9.6–25.6)
<10	136	(27.2)
10–20	197	(39.3)
>20	168	(33.5)
ISUP Grade		
1	17	(3.4)
2	112	(22.4)
3	201	(40.1)
4	96	(19.2)
5	75	(15.0)
Clinical T-stage		
T2	335	(66.9)
T3,4	166	(33.1)
NCCN risk group		
Intermediate	164	(32.7)
High	337	(67.3)
Perineural invasion		
Absent	260	(51.9)
Present	236	(47.1)
Inevaluable	5	(1.0)

Data are n (%) unless otherwise stated. Percentages may not total 100 due to rounding. Abbreviations: IQR, interquartile range; PSA, prostate-specific antigen; ISUP, International Society of Urological Pathology; NCCN, National Comprehensive Cancer Network.

**Table 2:** CCR2 * expression in prostate cancer biopsies—univariable and multivariable analysis of overall survival.

	Univariable (N = 501)			Multivariable (N = 492) †		
Endpoints	n	HR (95% CI)	*p*	n	HR (95% CI)	*p*
PSA progression	222	1.00 (0.97–1.04)	0.87	220	1.00 (0.96–1.04)	0.96
Bone progression	107	0.99 (0.94–1.04)	0.67	106	1.00 (0.95–1.06)	0.94
Distant progression	140	1.00 (0.95–1.04)	0.89	139	1.01 (0.97–1.05)	0.68
PC-specific mortality	64	0.98 (0.92–1.05)	0.58	64	0.99 (0.93–1.06)	0.86
All-cause mortality	174	0.98 (0.94–1.03)	0.44	172	0.98 (0.94–1.02)	0.38

* CCR2 H-score rescaled by dividing by 10; † 9 patients did not have RT dose as prescribed (7 had no RT, 1 had 50 Gy, and 1 had 76 Gy).

**Table 3:** Clinicopathological characteristics of patients used for CCL2 detection (n = 314).

Characteristic		
Age, median (IQR), years	68.7	(63.7–72.5)
PSA, median (IQR), ng/mL	15.5	(9.9–24.4)
<10	79	(25.2)
10–20	133	(42.4)
>20	102	(32.5)
ISUP Grade		
1	8	(2.6)
2	78	(24.8)
3	141	(44.9)
4	29	(9.2)
5	58	(18.5)
Clinical T-stage		
T2	206	(65.6)
T3,4	108	(34.4)
NCCN risk group		
Intermediate	110	(35.0)
High	204	(65.0)
Perineural invasion		
Absent	145	(46.2)
Present	166	(52.9)
Inevaluable	3	(1.0)

Data are n (%) unless otherwise stated. Percentages may not total 100 due to rounding. Abbreviations: IQR, interquartile range; PSA, prostate-specific antigen; ISUP, International Society of Urological Pathology; NCCN, National Comprehensive Cancer Network.

**Table 4:** CCL2 * expression in prostate cancer biopsies—univariable and multivariable analysis of overall survival.

	Univariable (N = 314)			Multivariable (N = 307) †		
Endpoints	n	HR (95% CI)	*p*	n	HR (95% CI)	*p*
PSA progression	134	1.03 (0.99–1.07)	0.09	132	1.03 (0.97–1.08)	0.36
Bone progression	71	1.03 (0.98–1.09)	0.26	70	1.03 (0.96–1.11)	0.36
Distant progression	92	1.03 (0.98–1.08)	0.25	91	1.03 (0.96–1.10)	0.41
PC-specific mortality	44	1.00 (0.90–1.11)	0.97	43	1.00 (0.88–1.13)	0.99
All-cause mortality	111	0.99 (0.93–1.06)	0.81	109	0.99 (0.93–1.06)	0.83

* CCL2 H-score rescaled by dividing by 10; † 7 patients did not have RT dose as prescribed (5 had no RT and 2 had 76 Gy).

**Table 5:** Clinicopathological characteristics of patient samples assayed in CCL2 ELISA (n = 220).

Characteristic		
Age, median (IQR), years	68.2	(62.9–73.0)
PSA, median (IQR), ng/mL	14.0	(8.8–23.0)
<10	67	(30.5)
10–20	89	(40.5)
>20	64	(29.1)
ISUP Grade		
1	10	(4.6)
2	60	(27.3)
3	59	(26.8)
4	48	(21.8)
5	43	(19.6)
Clinical T-stage		
T2	105	(47.7)
T3,4	115	(52.3)
NCCN risk group		
Intermediate	60	(27.3)
High	160	(72.7)
Perineural invasion		
Absent	140	(63.6)
Present	78	(35.5)
Inevaluable	2	(0.9)

Data are n (%) unless otherwise stated. Percentages may not total 100 due to rounding. Abbreviations: IQR, interquartile range; PSA, prostate-specific antigen; ISUP, International Society of Urological Pathology; NCCN, National Comprehensive Cancer Network.

**Table 6:** CCL2 quantification in serum of prostate cancer patients—univariable and multivariable analysis of overall survival.

	Univariable (N = 220)			Multivariable (N = 218) †		
Endpoints	n	HR (95% CI)	*p*	n	HR (95% CI)	*p*
PSA progression	90	1.50 (1.07–2.09)	0.017	90	1.19 (0.83–1.69)	0.35
Bone progression	49	1.20 (0.63–2.27)	0.58	49	0.91 (0.48–1.75)	0.78
Distant progression	69	1.44 (0.91–2.30)	0.12	69	1.10 (0.65–1.86)	0.72
PC-specific mortality	30	1.17 (0.53–2.60)	0.70	30	0.72 (0.31–1.63)	0.43
All-cause mortality	86	0.86 (0.48–1.54)	0.61	85	0.76 (0.40–1.44)	0.40

† 2 patients excluded as they did not have RT.

## Data Availability

The data presented in this study are available on request to the corresponding author.

## Supplementary Materials

The following supporting information can be downloaded at: https://www.mdpi.com/article/10.3390/cancers16162794/s1, Figure S1: Correlation between the three cohorts CCL2 serum, CCL2 tissue, and CCR2 tissue.
