# Supplementary material for: CCL2/CCR2 Expression in Locally Advanced Prostate Cancer and Patient Long-Term Outcome: 10-Year Results from the TROG 03.04 RADAR Trial
Source: Cancers (Basel). 2024 Aug 8;16(16):2794. doi: 10.3390/cancers16162794 (PMC11352466; doi:10.3390/cancers16162794)
Supplement: Supplementary file 1 [file cancers-16-02794-s001.zip › cancers-3116478-supplementary.pdf]

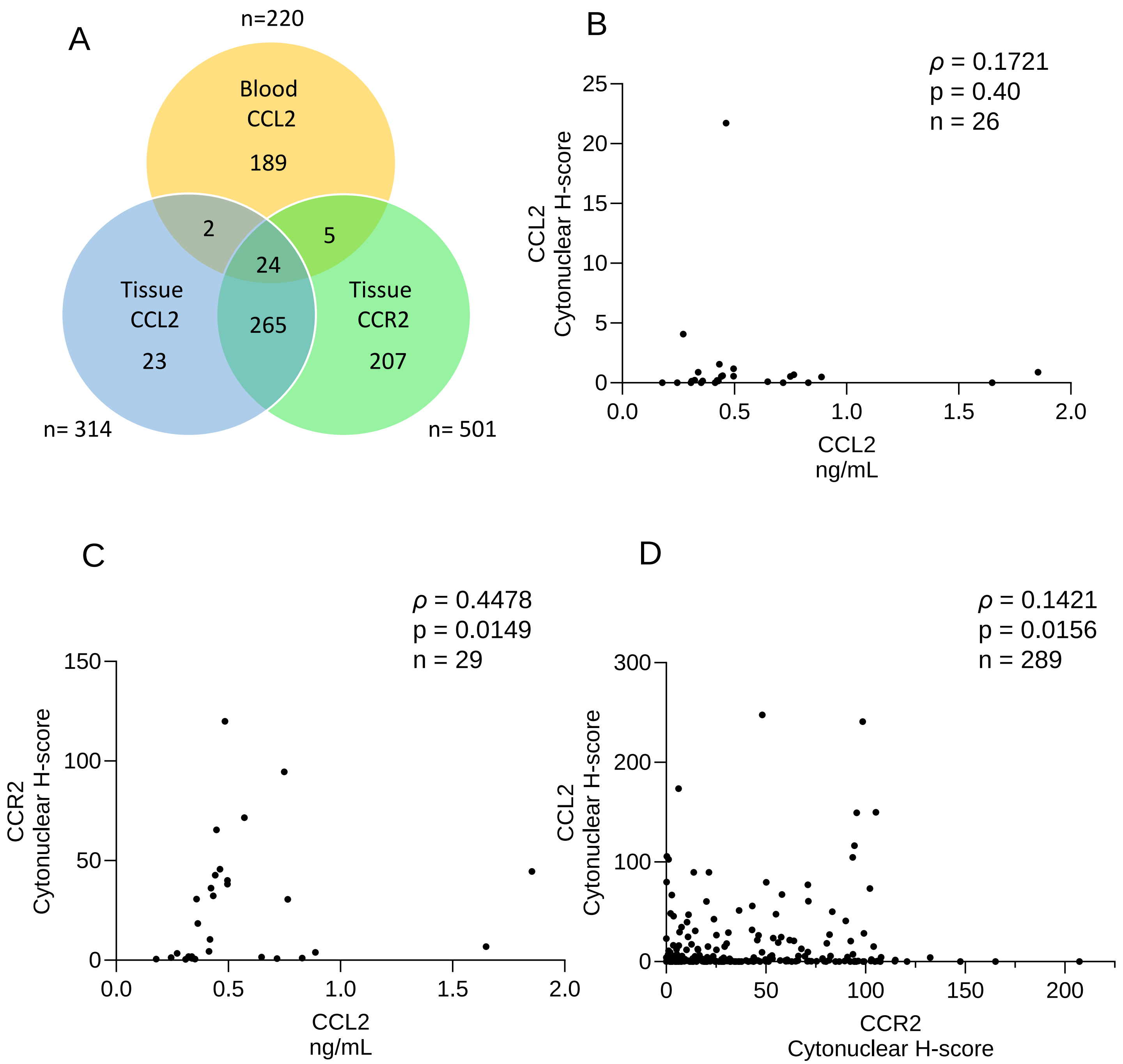

Figure S1. Correlation between the three cohorts CCL2 serum, CCL2 tissue and CCR2 tissue. A) Venn diagram showing the relationship between the three cohorts. B) Correlation between the CCL2 tissue H-score and CCL2 serum concentration. C) Correlation between the CCR2 tissue H-score and CCL2 serum concentration. D) Correlation between the CCR2 tissue H-score and CCL2 tissue H-score.
